# Retraction

**DOI:** 10.1111/jcmm.17601

**Published:** 2022-11-02

Retraction: Yang, C, Gu, Z, Ding, R, et al. Long non‐coding RNA MEG3 silencing and microRNA‐214 restoration elevate osteoprotegerin expression to ameliorate osteoporosis by limiting TXNIP. *J Cell Mol Med*. 2021; 25: 2025–2039. doi:10.1111/jcmm.16096.

The above article published online on 03 January 2021 in Wiley Online Library (wileyonlinelibrary.com) has been retracted by agreement between the authors, the journal Editor in Chief, and John Wiley and Sons Ltd. The retraction has been agreed following an investigation based on concerns raised by the authors and allegations raised by a third party. Several flaws and inconsistencies between results presented and experimental methods described were found, the editors consider the conclusions of this article to be invalid.

## References

[1] Yang C , Gu Z , Ding R , et al. Long non‐coding RNA MEG3 silencing and microRNA‐214 restoration elevate osteoprotegerin expression to ameliorate osteoporosis by limiting TXNIP. J Cell Mol Med. 2021;25:2025–2039. 10.1111/jcmm.16096. 33393160 PMC7882928

